# Association between apolipoprotein E gene polymorphism and nonalcoholic fatty liver disease in Southern China: A case‐control study

**DOI:** 10.1002/jcla.24061

**Published:** 2021-10-18

**Authors:** Sudong Liu, Ruiqiang Weng, Xiaodong Gu, Lihai Li, Zhixiong Zhong

**Affiliations:** ^1^ Center for Precision Medicine Meizhou People's Hospital (Huangtang Hospital) Meizhou China; ^2^ Provincial Key Laboratory of Precision Medicine and Clinical Translational Research of Hakka Population Meizhou China; ^3^ Research Experiment Center Meizhou People's Hospital (Huangtang Hospital) Meizhou China

**Keywords:** apolipoprotein E (ApoE), gene polymorphism, nonalcoholic fatty liver disease (NAFLD), Southern China

## Abstract

**Background:**

Apolipoprotein E (ApoE) polymorphisms have been reported to be associated with nonalcoholic fatty liver disease (NAFLD), but the conclusions of studies are inconsistent in different regions. The present study aims to investigate the role of ApoE genotypes on NAFLD in southern China.

**Methods:**

A total of 1064 subjects including 372 NAFLD patients and 692 controls who attended Meizhou People's Hospital located in southern China from March 1, 2016 to April 30, 2020 were enrolled in this study. The ApoE genotypes were detected and the laboratory parameters were examined.

**Results:**

Significant differences were observed between NAFLD patients and controls in the prevalence of ε3/ε3 (*p* < 0.001) and ε3/ε4 (*p* = 0.004). NAFLD patients presented higher frequency of ε4 allele than controls (*p* = 0.013). Logistic regression analysis suggested that ε3/ε3 was an independent risk factor (OR: 1.435, 95% CI: 1.084–1.891, *p* = 0.010), while ε3/ε4 was an independent protective factor (OR: 0.578, 95% CI: 0.404–0.828, *p* = 0.003) for development of NAFLD. In addition, allele ε4 showed a protective effect on NAFLD with an adjusted OR of 0.588 (95% CI: 0.420–0.824, *p* = 0.002).

**Conclusion:**

Our results suggested that ApoE genotype was associated with the development of NAFLD in the population of southern China. Individuals carrying ε3/ε3 were at higher risk of NAFLD, while those carrying ε3/ε4 were at lower risk of NAFLD.

## Graphical abstract

The study was aim to reveal the relationship between Apolipoprotein E gene polymorphism and nonalcoholic fatty liver disease in Southern China. The result showed that a significant differences were observed between NAFLD patients and controls in the prevalence of ε3/ε3 (P < 0.001) and ε3/ε4 (P = 0.004). NAFLD patients presented higher frequency of ε4 allele than controls (P = 0.013). Logistic regression analysis suggested Individuals carrying ε3/ε3 were at higher risk of NAFLD, while those carrying ε3/ε4 were at lower risk of NAFLD.

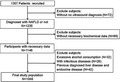

## 1 INTRODUCTION

Nonalcoholic fatty liver disease (NAFLD) refers to the exception of excessive drinking and other clear liver damage factors caused by fat deposition in liver cells, including simple liver steatosis, non‐alcoholic steatohepatitis (NASH), and cirrhosis. NAFLD is known to cause liver disability, and has been reported to associate with the high incidence of metabolic syndrome (MetS), type 2 diabetes mellitus (T2DM), arteriosclerotic cardiovascular disease, and colorectal tumors.^1^, ^2^ Studies suggest that the incidence of NAFLD is approximately 25%, but varied in different regions and population.^3^, ^4^ In the United States, about 25% of adults develop NAFLD, and one‐quarter of them may result in NASH, which increases the risk of liver cirrhosis and liver cancer.^4^, ^5^ In China, the prevalence of NAFLD ranges from 19% to 40% depending on regions.^6^ In southern China, the incidence is estimated to be about 28.83%, slightly higher than the global level.^7^ The variation of the NAFLD incidences is probably due to the sample size, modalities used for diagnosis, and diversity of lifestyles and dietary habits in different regions. Recent studies reveal that NAFLD was associated with lipid disorders.^8^, ^9^, ^10^ The disruption of the lipid metabolism balance in the liver leads to lipid accumulation, which in turn causes liver toxicity and NAFLD.^11^

Dyslipidemia is characterized by elevated levels of free fatty acids, low‐density lipoprotein‐cholesterol (LDL‐c) and triglycerides (TG), or reduced levels of high‐density lipoprotein‐cholesterol (HDL‐c). Dyslipidemia increases the deposition of fat in the liver, resulting in inflammation, lipotoxicity, and liver damage, which consequently contributes to the occurrence of NAFLD.^12^, ^13^ Apolipoprotein E (ApoE) is a plasma protein that plays an important role in dyslipidemia. The *ApoE* gene has 3 common alleles (ε2, ε3, ε4) which produce three homozygous (ε2/ε2, ε3/ε3, ε4/ε4) and three hybrid zygote (ε2/ε3, ε2/ε4, ε3/ε4) genotypes, as well as three isomers including ApoE2 (ε2/ε2, ε2/ε3), ApoE3 (ε3/ε3, ε2/ε4), and ApoE4 (ε3/ε4, ε4/ε4).^14^ Studies have shown that ApoE protein participates in the uptake of serum lipid by cells, and affects the metabolism of cholesterol and TG.^15^, ^16^ Previous studies have suggested that ε4 allele is a genetic risk factor for the development of liver disease, and individuals carrying ε4 are susceptible to NAFLD.^16^, ^17^ ApoE genotypes are correlated with serum LDL‐c levels, manifesting as an increased level of LDL‐c in person with ε4 allele and a decreased level in person with ε2 allele.^18^

Although some studies have investigated the relationship between ApoE genotypes and the incidence of NAFLD, the conclusions varied intensively.^16^ Moreover, little is known about the effect of ApoE polymorphism on the development of NAFLD in southern China. In the present study, we investigated the ApoE genotype distribution as well as lipid profiles in NAFLD patients from southern China and purposed to identify genetic factors associated with the development of NAFLD.

## 2 PATIENTS AND METHODS

### 2.1 Ethics statement

This study was performed in accordance with ethical standards specified by the Declaration of Helsinki and its amendments. It was approved by the Ethics Committee of Meizhou People's Hospital (NO: MPH HEC 2020‐C‐103).

### 2.2 Methods and demographic information

**FIGURE 1:**
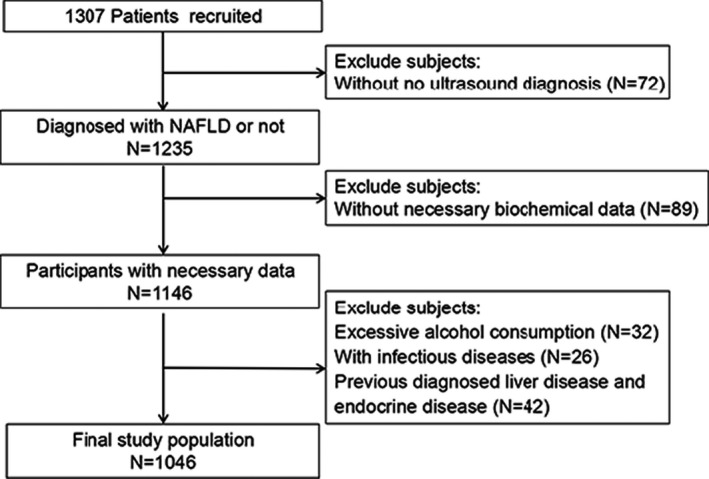
Flow diagram of selection of cases and controls from the cohort

General data: Three hundred and seventy‐two patients diagnosed with NAFLD in Meizhou People's Hospital from March 1, 2016 to April 30, 2020 were selected as the research subjects. The diagnosis of NAFLD was made according to the ultrasonic diagnostic criteria of fatty liver revised by the 2018 edition of the Chinese Medical Association guideline for the diagnosis and treatment of NAFLD.^19^ Six hundred and ninety‐two subjects with ultrasonographically normal livers during this period were recruited and served as controls. Patients with the following conditions were excluded from this study: (i) previous viral hepatitis, drug‐induced liver injury, hepatolenticular degeneration, autoimmune liver disease, or liver cancer; (ii) patients with history of alcoholism (alcohol consumption for men is > 30 g/d and for women > 20 g/d) or drug abuse; (iii) incomplete clinical data. A total of 372 NAFLD patients and 692 controls were finally included in this case‐control study (see selection diagram in Figure 1). Patients voluntarily participated in the study and signed the written informed consent.

The demographic information was collected by a general information questionnaire (including general demographic data, smoking history, drinking history, medical history, and medication history) conducted by doctors with unified training. The diagnosis of fatty liver was completed by a specialist using a B ultrasonic detector in the hospital and two physicians conformed the ultrasonic results.

### 2.3 Laboratory parameters measurement

After fasting for 8–12 h, 5 ml of venous blood was collected from the patient. The serum lipid profiles and blood glucose were examined by AU5400 analyzer (Beckman Coulter). The concentration of HbA1c was examined by Premier Hb9210 HbA1c Analytical Column (Trinity Biotech). The liver enzymes, including aspartate aminotransferase (AST), alanine aminotransferase (ALT), alkaline phosphatase (ALP), and glutamyltransferase (GGT), were measured using commercially available assays by AU5821 analyzer (Beckman Coulter).

### 2.4 *ApoE* genotyping

The DNA was extracted from the venous blood by Blood Genomic DNA Extraction Kit (Tiangen) and quantified by NanoDrop 2000 spectrophotometer (ThermoFisher). The ApoE genotyping was conducted using a commercially available kit (Sinochips Bioscience Co., Ltd). Briefly, samples were amplified on the Thermo Cycler (Life Technologies) with the following program: 50°C for 2 min, pre‐denaturation at 95°C for 15 min, 45 cycles of denaturing at 94 °C for 30 s, and annealing at 65°C for 45 s. The amplified products were analyzed by the fully automated GeneChip detection system.^20^

### 2.5 Statistical analysis

The Hardy‐Weinberg equilibrium was verified for all of the recruited patients. Data were analyzed by SPSS Statistics version 20.0 software (IBM). Continuous data were presented as mean ± SD and compared using Student's *t* test or Mann‐Whitney U‐test. Categorical variables were presented as number (%) and compared using chi‐square (χ^2^) test or Fisher's exact test. The logistic regression analysis was used to analyze the correlation between ApoE genotypes and risk of NAFLD. Adjusted odds (OR) were calculated to show the predictive value. All statistical tests were two‐sided and a *p* < 0.05 was considered significant.

## 3 RESULTS

### 3.1 Characteristics and laboratory features of study subjects

The characteristics and laboratory features of NAFLD patients and controls are presented in Table 1. A total of 372 NAFLD patients and 692 controls were included in the present study. The mean age was similar between groups, but the gender and DM patient distribution were different. The levels of TG, total cholesterol (TC), LDL‐c, apolipoprotein B (Apo B), and GGT were significantly higher in NAFLD compared with those in controls (all *p* < 0.05). The NAFLD patients had lower level of HDL‐c than controls (*p* < 0.01). In addition, the NAFLD patients had higher levels of ALT and AST than the controls (*p* < 0.01).

**TABLE 1:** Characteristic and laboratory features of NAFLD patients and controls

Variables	Total (n = 1064)	Controls (n = 692)	NAFLD (n = 372)	*p* value
Age (years)	60.18 ± 8.59	60.40 ± 8.39	59.77 ± 8.94	0.263
Gender (M/F)	715/349	481/211	234/138	0.029
BMI (kg/m^2^)	26.37 ± 6.3	25.4 ± 5.6	27.9 ± 4.6	<0.001
Diabetes mellitus	414 (38.91%)	233 (33.67%)	181(48.65%)	<0.001
HbA1c (%)	6.96 ± 1.84	6.75 ± 1.79	7.35 ± 1.87	<0.001
Glucose (mmol/L)	6.35 ± 2.95	6.11 ± 2.83	6.78 ± 3.09	<0.001
TG (mmol/L)	1.91 ± 1.63	1.66 ± 1.31	2.37 ± 2.03	<0.001
TC (mmol/L)	4.93 ± 1.35	4.85 ± 1.38	5.07 ± 1.29	0.011
HDL‐c (mmol/L)	1.19 ± 0.29	1.21 ± 0.31	1.14 ± 0.25	<0.001
LDL‐c (mmol/L)	2.85 ± 0.85	2.80 ± 0.84	2.93 ± 0.87	0.020
Apo A1 (g/L)	1.11 ± 0.26	1.10 ± 0.27	1.10 ± 0.24	0.832
Apo B (g/dl)	0.94 ± 0.29	0.92 ± 0.29	0.97 ± 0.29	0.005
ALT (U/L)	27.77 ± 21.81	25.69 ± 21.41	31.61 ± 22.64	<0.001
AST (U/L)	24.75 ± 14.73	24.35 ± 15.31	25.47 ± 13.58	0.240
ALP (U/L)	84.05 ± 38.87	84.72 ± 44.35	82.78 ± 28.24	0.438
GGT (U/L)	35.32 ± 38.54	32.15 ± 35.44	41.26 ± 43.15	<0.001
ALT/AST	1.11 ± 0.46	1.04 ± 0.45	1.23 ± 0.43	<0.001

Note

*p* value: the comparisons were made between NAFLD patients and controls using Student's *t* test or chi‐square (χ^2^) test.

Abbreviations: ALP, alkaline phosphatase; ALT, alanine aminotransferase; Apo A1, apolipoprotein A1; ApoB, apolipoprotein B; AST, aspartate aminotransferase; GGT, glutamyltransferase; HbA1c, glycated hemoglobin; HDL, high‐density lipoprotein; LDL, low‐density lipoprotein; TC, total cholesterol; TG, triglyceride.

### 3.2 Distribution of ApoE alleles and genotypes

As shown in Table 2, distribution of ApoE allele and genotypes in all subjects was reported. ApoE gene distribution in NAFLD and controls was consistent with Hardy‐Weinberg equilibrium (χ^2^ = 0.91, *p* = 0.923, χ^2^ = 2.56, *p* = 0.633 and χ^2^ = 4.323, *p* = 0.363, respectively). It was observed that compared with controls, NAFLD patients were more prevalent in the genotype of ε3/ε3 (67.74% *vs*. 61.84%, *p* < 0.001) and ε2/ε2 (1.61% *vs*. 0.43%, *p* = 0.045), while less prevalent in the genotype of ε3/ε4 (13.71% *vs*. 23.95%, *p* = 0.004). There were no significant differences in NAFLD patients and controls with the genotype of ε2/ε3 (13.87% *vs*. 13.98%, *p* = 0.962), ε2/ε4 (1.61% *vs*. 1.58%, *p* = 0.977) and ε4/ε4 (1.34% *vs*. 1.30%, *p* = 0.762).

**TABLE 2:** Genotype distributions and allele frequencies in NAFLD patients and controls

Variables	Total	Control (n = 692)	NAFLD (n = 372)	*p* value
Genotype				
ε2/ε2	9 (0.84%)	3 (0.43%)	6 (1.61%)	0.045
ε2/ε3	148 (13.90%)	96 (13.87%)	52 (13.98%)	0.962
ε2/ε4	17 (1.60%)	11 (1.58%)	6 (1.61%)	0.977
ε3/ε3	680 (63.91%)	428 (61.84%)	252 (67.74%)	< 0.001
ε3/ε4	196 (18.42%)	145 (20.95%)	51 (13.71%)	0.004
ε4/ε4	14 (1.31%)	9 (1.30%)	5 (1.34%)	0.762
Allele				
ε2	183 (8.60%)	113 (8.17%)	70 (9.41%)	0.329
ε3	1704 (80.08%)	1097 (79.26%)	607 (81.59%)	0.201
ε4	241 (11.32%)	174 (12.57%)	67 (9.00%)	0.013
Phenotype				
ApoE2	157 (14.75%)	99 (14.31%)	58 (15.59%)	0.573
ApoE3	697 (63.82%)	439 (63.44%)	258 (69.35%)	0.053
ApoE4	210 (19.73%)	154 (22.25%)	56 (15.06%)	0.005
HWE	Χ^2^ = 0.910, *p* = 0.923	Χ^2^ = 2.561, *p* = 0.633	Χ^2^ = 4.323, *p* = 0.363	

Note

*p* value: comparisons were made between NAFLD patients and controls using chi‐square (χ^2^) test.

Abbreviations: NAFLD, Nonalcoholic fatty liver disease; HWE, Hardy‐Weinberg equilibrium.

### 3.3 Effect of ApoE on lipid profile

We next investigated the effect of ApoE genotype on serum lipid profile. Previous studies have shown the opposite effects of ε2 and ε4 allele on lipid metabolism, so the ε2/ε4 phenotype was excluded from the analysis.^14^ The subjects were divided into three subgroups based on their phenotype, namely ApoE2 (ε2/ε2 + ε2/ε3), ApoE3 (ε3/ε3), and ApoE4 (ε4/ε3 + ε4/ε4). The comparisons of serum lipid‐lipoprotein levels and liver function index between NAFLD patients and controls in subgroups were shown in Table 3. It was observed that TG levels were significantly higher in NAFLD patients than those in controls in all subgroups (all *p* < 0.05). In the subgroup of ApoE3, NAFLD patients exhibited significantly lower HDL‐c and higher LDL‐c than controls. In the subgroup of ApoE2 and ApoE3, NAFLD patients had higher ApoB than controls.

**TABLE 3:** Relationship between serum lipid‐lipoprotein levels and ApoE phenotype in NAFLD patients and controls

Variables	ApoE2 (ε2/ε2 + ε2/ε3)		ApoE3 (ε3/ε3)		ApoE4 (ε4/ε3+ε4/ε4)	
	Control (n = 99)	NAFLD (n = 58)	Control (n = 439)	NAFLD (n = 258)	Control (n = 154)	NAFLD (n = 56)
HbA1c (%)	6.80 ± 1.97	7.22 ± 1.60	6.76 ± 1.77	7.43 ± 1.95**	6.74 ± 1.78	7.23 ± 1.75
Glucose (mmol/L)	6.55 ± 2.91	7.05 ± 4.35	6.02 ± 2.83	6.65 ± 2.66**	6.17 ± 2.85	7.11 ± 3.48*
TG (mg/dl)	1.27 ± 1.01	2.32 ± 1.72**	1.65 ± 1.38	2.09 ± 1.62*	1.17 ± 1.17	2.52 ± 3.25**
TC (mg/dl)	4.18 ± 1.30	4.67 ± 1.36*	4.85 ± 1.35	4.99 ± 1.20	4.51 ± 1.21	4.76 ± 1.38
HDL‐c (mg/dl)	1.19 ± 0.36	1.13 ± 0.27	1.23 ± 0.31	1.15 ± 0.25**	1.19 ± 0.30	1.13 ± 0.27
LDL‐c (mg/dl)	2.64 ± 0.83	2.80 ± 0.71	2.79 ± 0.82	2.92 ± 0.83*	2.97 ± 0.88	3.04 ± 1.01
Apo A1 (mg/dl)	1.08 ± 0.24	1.12 ± 0.25	1.12 ± 0.28	1.10 ± 0.23	1.07 ± 0.26	1.10 ± 0.26
Apo B (mg/dl)	0.85 ± 0.24	0.93 ± 0.25*	0.91 ± 0.28	0.96 ± 0.27*	0.99 ± 0.32	1.02 ± 0.34
ALT (U/L)	25.96 ± 16.08	32.1 5 ± 18.62*	26.40 ± 24.62	30.31 ± 22.20*	23.12 ± 13.30	36.58 ± 24.80**
AST (U/L)	23.19 ± 8.49	27.84 ± 19.95*	24.40 ± 17.66	24.35 ± 11.74	24.52 ± 11.44	27.83 ± 13.20
ALP (U/L)	87.37 ± 29.01	81.62 ± 26.20	84.54 ± 29.13	83.11 ± 29.11	83.84 ± 75.19	81.35 ± 26.62
GGT(U/L)	36.08 ± 35.69	43.37 ± 42.97	32.88 ± 39.71	38.00 ± 30.16	27.52 ± 20.00	51.71 ± 78.41**
ALT /AST	1.14 ± 0.66	1.17 ± 0.36	1.05 ± 0.41	1.22 ± 0.45**	0.99 ± 0.40	1.29 ± 0.43**

Note

*p < 0.05, **p < 0.01: comparisons were made between patients and controls in the same ApoE phenotype group using Student's *t* test.

Abbreviations: ALP, alkaline phosphatase; ALT, alanine aminotransferase; Apo A1, apolipoprotein A1; ApoB, apolipoprotein B; AST, aspartate aminotransferase; GGT, glutamyltransferase; HbA1c, glycated hemoglobin; HDL, high‐density lipoprotein; LDL, low‐density lipoprotein; TC, total cholesterol; TG, triglyceride.

We also analyzed the liver enzyme between NAFLD patients and controls in different subgroups. It was observed that NAFLD patients presented significantly higher level of ALT (*p* < 0.05) in all of subgroups, higher level of AST in the subgroup of ApoE2 (*p* < 0.05), and higher level of GGT in the subgroup of ApoE4 (*p* < 0.01). In addition, the ratio of ALT/AST was higher than the controls in the subgroup of ApoE3 and ApoE4 (*p* < 0.001).

### 3.4 Risk factors for NAFLD

Logistic regression analysis was applied to determine the predicting value of ApoE genotype and allele for NAFLD. As shown in Table 4, it was indicated that ε3/ε3 was a risk factor for NAFLD (adjusted OR: 1.435, 95% CI: 1.084–1.891, *p* = 0.010), while ε3/ε4 was a protective factor for NAFLD (adjusted OR: 0.578, 95% CI: 0.404–0.828, *p* = 0.003). In addition, allele ε4 showed a protective effect in the development of NAFLD (adjusted OR: 0.588, 95% CI: 0.420–0.824, *p* = 0.002). The clinical studies of the research addressing ApoE gene polymorphisms and NAFLD are summarized in Table 5. In different regions, the association between ApoE gene polymorphism and NAFLD was different. In the present study, we found ApoE ε3/ε3 genotype acted as an independent risk factor of NAFLD, and ε3/ε4 was played as a protective factor in the development of NAFLD for population in southern China.

**TABLE 4:** Logistic regression analysis of risk factors for NAFLD

Genotype	Adjusted OR	95% CI	*p* value
ε2/ε2	2.401	0.575–10.022	0.230
ε2/ε3	0.956	0.659–1.389	0.815
ε3/ε3	1.435	1.084–1.891	0.010
ε3/ε4	0.578	0.404–0.828	0.003
ε4/ε4	0.630	0.175–2.269	0.479
ε2/ε4	0.728	0.244–2.173	0.569
Allele ε2	0.983	0.693–1.395	0.925
Allele ε3	0.973	0.491–1.929	0.937
Allele ε4	0.588	0.420–0.824	0.002

Note

Adjusted OR: adjusted by BMI, diabetes mellitus and TG.

**TABLE 5:** Studies of ApoE polymorphism on NAFLD in humans

Authors	Region	Studies characteristics	Allele frequencies	Outcome
Emma De Feo et al.^29^	Rome, Italy	310 NAFLD patients and 422 controls	↑ε3 ↓ε4	NAFLD risk
Ali Sazci et al.^25^	Kocaeli, Turkey	57 NASH patients and 245 controls	↑ε3, ε3/ε3, ↓ε4, ε2/ε4, ε2/ε3	NAFLD risk
O Chernyak et al.^26^	Moscow, Russia	22 NASH patients and 50 controls	↓ε3/ε4	Prognostic of NAFLD
Moon Hee Yang et al.^17^	Seoul, Korea	Cross‐sectional study: 711 NAFLD patients and 711 controls	↑ε4	NAFLD risk
E Stachowska et al.^35^	Szczecin, Poland	Prospective study: 23 patients with NAFLD	↓ε3 ↑ε4	Risk of advanced fibrosis
Mehmet Derya Demirag et al.^27^	Ankara, Turkey	237 NAFLD patients and 201 controls	↑ε2, ε2/ε3	Protective against NAFLD
Present study	Guangdong, China	372 NAFLD patients and 692 controls	↑ε3/ε3 ↓ε4, ε3/ε4	NAFLD risk

Note

↑represents an increased allele frequency in NAFLD patients; ↓represents a decreased allele frequency in NAFLD patients.

## 4 DISCUSSION

Nonalcoholic fatty liver disease is widely considered as the most common cause of chronic liver diseases, and has become an important global public health problem.^5^ Recent studies have revealed that NAFLD was associated with lipid disorders and disruption of lipid metabolism balance in the liver.^1^ ApoE is a key protein that affects lipid metabolism, and its polymorphism has been reported to be associated with incidence of NAFLD in various populations. In the present study, we investigated the lipid profiles and ApoE genotype distribution in NAFLD patients and controls from southern China. We found that ApoE influenced the serum lipid profile in NAFLD patients, and ε3/ε3 served as independent risk factor, while ε3/ε4 and allele ε4 serve as protective factor of NAFLD.

Apolipoprotein E is a multifunctional protein whose synthesis, secretion, and metabolism are mainly completed in the liver. ApoE plays a key role in the metabolism of TC and TG.^18^ ApoE‐deficient mice fed with high‐fat diet intensively increased TG accumulation in the liver,^21^ while introduction of the ε3 transgene reversed this process.^22^ It has been reported that ApoE protein participated in the degradation of LDL, HDL, and other lipids by binding to its receptors, and this process depended on the affinity between ApoE and receptors.^23^ Study showed that ApoE protein of ε2 allele preserved low affinity with LDL receptor, and ApoE protein of ε4 allele had higher affinity with LDL receptors. As a result, ε2 allele carriers prone to have lower level of TC and LDL‐c, while ε4 allele carriers have high level of TC and LDL‐c.^24^ Consistently, we observed that the serum levels of TC and LDL‐c in ε2 NAFLD patients were lower than those in ε4 carrier.

Research revealed that ApoE gene played an important role in the pathogenesis of NAFLD.^16^ The relationship between ApoE gene polymorphism and NAFLD varied in different populations and regions. Sazci A. et al.^25^ performed a case‐control study in a cohort of 57 NAFLD patients and 245 healthy controls from Turkey population, and found that ε3/ε3 was a risk factor of NAFLD. In Russia population, the presence of ε3/ε4 was a poor prognostic marker of NAFLD.^26^ A study in Ankara consisting of 237 NAFLD patients and 201 controls suggested that ε2/ε3 genotype might be protective against the development of NAFLD.^27^ Ali Sazci et al.^25^ reported that patients with ε2/ε4 and ε2/ε3 might be at higher risk of developing NAFLD. More studies addressing ApoE polymorphisms and NAFLD were shown in Table 5. In consistent with previous study, we found that in southern China the ApoE ε3/ε3 was an independent risk factor of NAFLD. However, ε3/ε4 appeared to be a protective factor of NAFLD, which was different from outcomes in other studies. A potential reason for the discrepancy may be attributed to different ethnic, the lifestyle habits, and clinical characteristics.^28^ Otherwise, there was not any significant association between ε2/ε4 and ε2/ε3 with NAFLD in the present study. It was also observed that ε4 allele held a significantly protective effect on the development of NAFLD, which was in accord with findings from the previous studies.^29^

The mechanisms regarding how ApoE influences the etiopathogenesis of the NAFLD were not very clear, but evidences suggested that this process may be involved in lipid metabolism. Studies suggested that ApoE is involved in the regulation of hepatic TG‐rich VLDL secretion.^30^, ^31^ Kypreos et al.^32^ found the ApoE4 was essential for promoting hepatic TG‐rich VLDL secretion. It was indicative that ε4 allele may act as a protective factor of NAFLD in the regulation of TG‐rich VLDL rather than the cholesterolemia. Unlike ε4, ε3 preferentially targeted the HDL rather than VLDL, and alter the lipolytic processing in the circulation.^33^ In this regard, individuals carrying ε4/ε3 had a lower level of VLDL‐apoB than those with ε3/ε3.^30^ It also had a significant association between serum ApoE and lipid peroxide levels in ε4/ε3 phenotype patients.^34^

This was the first study to investigate the relationship between ApoE gene polymorphism and NAFLD in southern China. Some limitations should be noted. First, the sample size of the study group was not big enough, which might compromise the findings. Second, the present case‐control study was conducted limited in Meizhou, southern China, and the generalizability of the findings in other populations needs to be tested. Third, the fact that subjects were recruited from hospital might lead to selection bias.

## 5 CONCLUSION

In conclusion, the present study investigated the relationship between ApoE polymorphisms and NAFLD in southern China. Our data suggested that ε3/ε3 might serve as an independent risk factor, while ε3/ε4 and allele ε4 as a protective factor of NAFLD. Our findings may provide practical information for the prevention and treatment of NAFLD in this region.

## CONFLICT OF INTEREST

The authors declare that they have no competing interests.

## Data Availability

The datasets used and/or analysis during the current study available from the corresponding author on reasonable request.
